# Case Report: Hemolytic anemia secondary to infliximab treatment in a patient with ulcerative colitis

**DOI:** 10.3389/fmed.2025.1548321

**Published:** 2025-03-12

**Authors:** Gerolamo Bevivino, Patrizio Scarozza, Michela Di Fonzo, Giulia Zerboni, Federico Iacopini

**Affiliations:** Gastroenterology and Digestive Endoscopy Unit, Ospedale dei Castelli Hospital, Rome, Italy

**Keywords:** infliximab, hemolytic anemia, ulcerative colitis, autoimmune disorders, case report

## Abstract

Infliximab, a monoclonal antibody targeting tumor necrosis factor-alpha (TNF-α), is widely used in treating inflammatory bowel diseases (IBD), including ulcerative colitis (UC). While generally well-tolerated, infliximab is associated with rare but significant adverse effects, including autoimmune hemolytic anemia (AIHA). This report describes the case of a 54-year-old male diagnosed with UC, who developed hemolytic anemia secondary to infliximab therapy after 1 year of treatment. During the infusion preceding the onset of anemia, the patient experienced a severe infusion reaction characterized by urticaria, bronchospasm, chills, fever, and pulsating headache. Laboratory findings confirmed hemolytic anemia with a positive direct and negative indirect Coombs tests. The patient responded well to corticosteroid therapy (prednisone at 1 mg/kg/day for 30 days) and stopping anti-TNF-α, with hemoglobin levels improving from 7.2 g/dL at presentation to 14.6 g/dL after 1 month. AIHA should be considered an uncommon but serious complication of infliximab therapy, necessitating careful monitoring, especially in patients treated for gastrointestinal indications. This case underscores the importance of recognizing and managing infusion-related complications of biologic therapies.

## Introduction

Ulcerative colitis (UC) is a chronic inflammatory bowel disease (IBD) often treated with biological agents like infliximab (1). While effective for disease control, infliximab can cause rare but severe adverse events, such as infusion reactions and autoimmune hemolytic anemia (AIHA).

Infliximab has revolutionized the management of UC by reducing inflammation and inducing remission. However, it can trigger autoimmune phenomena, leading to conditions such as lupus-like syndrome and AIHA (2, 3). AIHA is characterized by the premature destruction of red blood cells due to autoantibodies, presenting challenges in diagnosis and management, especially in the context of IBD treatment.

This report highlights a case of AIHA following a severe infusion reaction, emphasizing the importance of early recognition, effective corticosteroid therapy, and close monitoring.

## Case description

A 54-year-old male was diagnosed with extensive UC in March 2022. His medical history included:

- Hypercholesterolemia, treated with atorvastatin (20 mg/day).
- Arterial hypertension, managed with zofenopril calcium/hydrochlorothiazide (30 mg/12.5 mg daily).
- Type II diabetes mellitus (T2DM), controlled with metformin (1,000 mg twice daily).
- History of resolved HBV infection, with undetectable HBV-DNA and positive anti-HBs antibodies.
- History of anemia, with mild chronic anemia (Hb ranging between 11.5–12.5 g/dL) noted since UC diagnosis, likely due to chronic inflammation and episodic gastrointestinal blood loss. The patient required iron supplementation intermittently but had never required blood transfusions or corticosteroids for anemia prior to infliximab initiation.

The patient is a non-smoker and does not consume alcohol or illicit drugs. He works as an accountant and lives with his spouse. No recent travel history or known occupational exposures. No known autoimmune disorders, hematologic diseases, or inflammatory bowel disease in first-degree relatives.

The patient was afebrile, hemodynamically stable, and had an unremarkable physical examination. No jaundice, lymphadenopathy, hepatosplenomegaly, or signs of acute distress were observed.

The patient was initially managed with mesalamine (4.8 g/day) and corticosteroids. Due to chronic active UC, infliximab was started in December 2022, achieving clinical and endoscopic remission after 1 year.

### Infusion reaction

During the fifth maintenance infliximab infusion, the patient experienced a severe infusion reaction characterized by fever, preceded by chills, urticaria with diffuse, itchy rash, pulsating headache, and bronchospasm with wheezing and mild respiratory distress.

The infusion was immediately stopped, and the patient was treated with intravenous antihistamines, corticosteroids, and oxygen. Symptoms resolved within 2 h, and the patient was discharged.

### Onset of anemia

Two days after the infusion reaction, the patient experienced intense fatigue, dyspnea on exertion and diffuse peripheral arthralgias with joint stiffness. Laboratory tests revealed severe anemia with hemoglobin 7.2 g/dL (reference range: 13.5–17.0 g/dL), lactate dehydrogenase (LDH) 480 U/L (normal range: 140–220 U/L), mild hyperbilirubinemia (total bilirubin 2.0 mg/dL), and a markedly elevated reticulocyte count (26%). The direct Coombs test was positive for IgG and negative for C3, confirming the diagnosis of warm AIHA. The indirect Coombs test was negative, indicating the absence of preformed alloantibodies. Peripheral blood smear showed anisocytosis, polychromasia, and spherocytosis, confirming hemolysis.

### Management

Infliximab was discontinued, and prednisone at a dosage of 1 mg/kg for 30 days was administered. Folic acid supplementation was also implemented. Fatigue resolution and improvement of Hb levels were observed in 1 week, with normalization at 1 month of follow up (Hb = 14.6 g/dL).

Three months after the adverse reaction and discontinuation of infliximab, a colonoscopy was performed with lesions at descending colon level (Mayo endoscopic score = 2). Histological examination of all segments revealed mild ulcerative colitis activity (Nancy histological index = Grade 2).

The patient experienced several courses of low-bioavailability corticosteroids developing steroid-dependance and after 8 months, vedolizumab was initiated with benefit in short and long-term (4).

## Discussion

Severe infusion reactions to infliximab, such as fever, chills, urticaria, and bronchospasm, may precede immune dysregulation and hemolytic complications. This case demonstrates the importance of recognizing infusion-related hypersensitivity as a potential trigger for autoimmune processes.

Prompt corticosteroid therapy was highly effective in this case, leading to a rapid improvement in hemoglobin and clinical symptoms.

Regular monitoring of hemoglobin, reticulocyte count, and LDH levels is crucial during treatment.

AIHA is a rare but potentially severe adverse effect associated with infliximab therapy.

This report underscore a common theme: discontinuation of infliximab and transitioning to alternative biologics (e.g., vedolizumab) or immunomodulators can effectively control the underlying IBD while reducing the risk of autoimmune side effects. Clinicians should remain vigilant for AIHA symptoms during infliximab treatment, given its potential for rapid progression and severity.

Drug-induced hemolytic anemia (DIHA) is a rare condition characterized by the premature destruction of red blood cells (RBCs) due to adverse reactions to certain medications (5). This phenomenon can occur through various mechanisms, including immune-mediated processes where drugs induce the production of antibodies that target RBCs. One such mechanism involves the formation of autoantibodies that react with RBC antigens, leading to their destruction.

Hemolytic anemia is a rare but recognized extraintestinal manifestation of IBD. It occurs when the immune system mistakenly targets and destroys red blood cells, leading to hemolysis. The exact mechanism linking hemolytic anemia to IBD is not fully understood, but it is thought to involve immune dysregulation and chronic inflammation. IBD flares often coincide with the onset or worsening of hemolytic anemia, suggesting a direct relationship between these two conditions (6, 7).

Infliximab, a chimeric monoclonal antibody targeting tumor necrosis factor-alpha (TNF-α), is widely used in the treatment of autoimmune diseases such as rheumatoid arthritis, Crohn’s disease (CD), and UC. While generally effective, infliximab has been associated with adverse hematologic events, including the development of AIHA.

The exact pathogenesis of infliximab-induced AIHA is not fully understood. However, it is hypothesized that infliximab may disrupt immune tolerance, leading to the production of autoantibodies against RBCs. This disruption could result from the drug’s immunomodulatory effects, which may alter the balance of immune regulation and promote autoimmunity. As demonstrated in several case reports (8–14) monitoring anti-TNF agents administration is essential in patients with IBD (Table 1).

**Table 1:** Summary of reported cases of infliximab-associated autoimmune hemolytic anemia (AIHA) and AIHA as an extraintestinal manifestation of inflammatory bowel disease (IBD).

Study	Year	Disease (UC/CD)	AIHA onset	Direct Coombs (IgG/C3)	Management	Rechallenge
Vermeire et al. (2)	2003	CD	After 3 infusions	Negative	Steroids	No
					Discontinuation of infliximab	
Colombel et al. (8)	2004	UC/CD	Idiosyncratic	Positive IgG	Discontinuation of infliximab	No
Fidder et al. (9)	2009	UC/CD	After 1 year	Negative	Steroids	No
					Discontinuation of infliximab	
Mir et al. (12)	2017	UC	After 5 infusions	Positive IgG	Discontinuation of infliximab only	No
Toplicanin et al. (13)	2022	CD	After 6 infusions	Positive IgG	Steroids	Yes (dose escalation before switch)
Strik et al. (14)	2019	CD	After switching to biosimilar	Negative	Steroids	No
					Discontinuation of infliximab	
Al-Ansari et al. (10)	2021	UC	After 4 infusions	Positive IgG	Steroids	No
					Discontinuation of infliximab	
Present case	2024	UC	After 1 year	Positive IgG, negative C3	Steroids	No
					Discontinuation of infliximab + vedolizumab	

This table summarizes documented cases of AIHA in patients with IBD receiving infliximab. The distinction between drug-induced immune hemolytic anemia (DIIHA) and IBD-related AIHA is highlighted, as infliximab has been reported both as a trigger of AIHA (DIIHA) and as a treatment for AIHA secondary to IBD. Key variables include the time of AIHA onset relative to infliximab exposure, the direct Coombs test results (IgG/C3), treatment approaches, and whether infliximab was discontinued or rechallenged. This synthesis provides clinicians with a comparative perspective on disease course, diagnostic considerations, and management strategies in patients with IBD and AIHA.

Infliximab-induced AIHA has been documented in multiple case reports, though its exact pathogenesis remains incompletely understood. The first prospective evidence of autoimmune complications linked to anti-TNF therapy was reported by Colombel et al. (8), who described various autoimmune manifestations, including hemolytic anemia, in patients treated with infliximab for IBD. Similarly, Fidder et al. (9) reviewed long-term safety data on infliximab therapy and identified cases of Coombs-negative hemolytic anemia, raising questions about whether infliximab-induced AIHA follows a purely immune-mediated mechanism in all cases.

More recently, Al-Ansari et al. (10) presented a case of severe AIHA following infliximab infusion in a UC patient, reinforcing the potential for infliximab to induce immune-mediated red blood cell destruction. Notably, their case, like ours, involved a positive direct Coombs test for IgG, consistent with warm AIHA, requiring steroid therapy and discontinuation of infliximab. The growing number of reports highlights the necessity of heightened awareness for hemolytic complications in IBD patients receiving biologics.

Vermeire et al. (11) investigated predictive factors for the response to infliximab and reported that three patients experienced hematological complications, including one case of AIHA.

Mir et al. (12) reported a case of infliximab-induced AIHA in a UC patient, highlighting the diagnostic challenges and the importance of early intervention with corticosteroids after infliximab therapy withdrawal. Toplicanin et al. (13) reported a case of a CD patient with recurrent AIHA that preceded infliximab therapy, suggesting that AIHA in this instance was likely secondary to the underlying IBD rather than directly infliximab-induced. The patient initially responded to high-dose steroids, but experienced relapses upon steroid tapering. Infliximab was later introduced to manage both CD and AIHA as an extraintestinal manifestation. During a subsequent AIHA relapse, low infliximab trough levels led to an increase in dosing frequency (from every 6 weeks to every 4 weeks). At a later relapse, the presence of anti-infliximab antibodies prompted a switch to vedolizumab. Strik et al. (14) reported a patient with CD who developed hemolytic anemia 6 months after switching from IFX originator to IFX biosimilar (CT-P13), reinforcing the need for alternative therapies in managing both IBD and associated autoimmune complications.

The management of infliximab-induced AIHA should follow evidence-based recommendations for drug-induced immune hemolytic anemia (DIHA), as outlined by the British Society for Haematology Guidelines (15). These guidelines emphasize that drug cessation is the first-line intervention, with steroids commonly used but of uncertain benefit due to difficulty in differentiating their effect from that of drug discontinuation. In non-severe cases, cessation of infliximab alone may be sufficient for hematologic recovery within 1–2 weeks, as demonstrated by Mir et al. (12). However, in severe cases, management should include fluid resuscitation, close monitoring of renal function, and transfusional support, with IVIG or plasmapheresis as adjunctive therapies if necessary. The real-world use of corticosteroids remains widespread, despite limited evidence from controlled studies supporting their efficacy in DIHA. In our case, steroid therapy (prednisone 1 mg/kg) led to a rapid hematologic response, with hemoglobin levels normalizing within 1 month. Close monitoring of hematologic parameters is essential to assess response to therapy and guide further treatment decisions.

Hemolytic anemia is a rare but recognized extraintestinal manifestation of IBD, and distinguishing autoimmune hemolytic anemia (AIHA) secondary to IBD from drug-induced immune hemolytic anemia (DIIHA) presents a diagnostic challenge. AIHA secondary to IBD is believed to result from chronic immune dysregulation and inflammation, which may trigger the production of autoantibodies against red blood cells. Notably, infliximab has been used as a treatment for IBD-associated AIHA, particularly in corticosteroid-refractory cases (8, 9). In contrast, infliximab-induced DIIHA is a direct consequence of the drug itself, either through immune complex formation, drug-dependent antibodies, or nonspecific immune activation.

AIHA secondary to IBD often develops independent of specific drug exposure, whereas DIIHA typically occurs after drug initiation or dose escalation.

In cases of AIHA secondary to IBD, infliximab may actually improve anemia due to its immunomodulatory effect, while in DIIHA, hemolysis worsens after infliximab exposure. Both conditions usually present with a positive direct Coombs test, but DIIHA may involve different antibody subtypes, such as drug-dependent IgG or IgM, whereas AIHA secondary to IBD often follows a warm AIHA pattern (IgG positive, C3 negative). In DIIHA, drug cessation is the primary treatment, often leading to hematologic improvement within 1–2 weeks (15). In contrast, AIHA secondary to IBD may require prolonged immunosuppressive therapy, and biologics such as infliximab or vedolizumab can be beneficial in refractory cases.

The case described by Toplicanin et al. (13) highlights this distinction, where AIHA preceded infliximab initiation and was managed with infliximab dose escalation before switching to vedolizumab due to anti-drug antibodies. In contrast, our case is more consistent with DIIHA, given the clear temporal association with infliximab exposure, the severe hemolytic reaction following an infusion reaction, and the rapid resolution upon infliximab discontinuation and corticosteroid therapy.

Recognizing these differences is crucial, as misattributing AIHA to infliximab when it is actually an IBD-related extraintestinal manifestation could lead to premature discontinuation of an effective treatment. Conversely, failing to recognize true DIIHA could result in continued drug exposure, worsening hemolysis, and potential complications.

Given the risk of recurrent AIHA with infliximab, transitioning to vedolizumab provided an alternative therapeutic option with sustained IBD control. However, clinicians should be aware that vedolizumab itself has been implicated in rare cases of hemolytic anemia (16). While our patient tolerated vedolizumab well, ongoing hematologic monitoring is advised in cases of biologic switching after AIHA.

The use of prophylactic corticosteroids during infliximab infusion is primarily recommended to reduce immediate infusion reactions rather than prevent delayed autoimmune side effects such as AIHA. The American College of Gastroenterology (ACG) and European Crohn’s and Colitis Organisation (ECCO) guidelines suggest premedication (antihistamines, corticosteroids) in patients with a history of infusion reactions to reduce hypersensitivity risk (17, 18). However, their role in preventing AIHA is not well established.

Our case showed a severe infusion reaction preceding the AIHA diagnosis, suggesting a potential link between immune activation and subsequent autoimmunity. While corticosteroid premedication could theoretically mitigate this risk, existing data are inconclusive. A systematic review found that steroid premedication reduces acute reactions but has no proven effect on long-term immune dysregulation (19). Moreover, cases of AIHA have been reported without prior infusion reactions, implying a distinct pathophysiological mechanism (12).

Thus, we do not advocate universal steroid premedication for AIHA prevention. However, for high-risk patients such as those with previous severe infusion reactions or underlying autoimmune predispositions individualized corticosteroid prophylaxis may be considered. This has been clarified in the discussion.

While this case highlights a temporal association between infliximab administration and the onset of AIHA, the causal relationship remains uncertain. AIHA is a known extraintestinal manifestation of IBD and has been reported in UC and CD, regardless of pharmacological treatment (20). Therefore, it is possible that the patient’s underlying disease, rather than infliximab itself, contributed to the development of AIHA.

Moreover, infliximab is widely used across multiple immune-mediated inflammatory diseases (IMIDs), including gastrointestinal, rheumatologic, and dermatologic conditions. Notably, no confirmed cases of infliximab-induced AIHA have been reported in patients treated for rheumatologic or dermatologic indications, despite millions of patients having been exposed to the drug worldwide over the years (2). This raises the possibility that the association between infliximab and AIHA may be more context-dependent rather than a direct drug-related effect.

Furthermore, prior studies suggest that hemolytic anemia has been reported in the setting of IBD both with and without exposure to pharmacologic treatment, reinforcing the need for a cautious interpretation of the causality between infliximab and AIHA (21). Notably, no positive rechallenge cases have been published where infliximab was successfully reintroduced following AIHA in IBD patients, further complicating the establishment of a definitive causality. Given the availability of multiple therapeutic alternatives for IBD, clinicians often opt for switching biologics rather than attempting infliximab re-exposure in such cases.

The management of IFX-induced AIHA typically involves drug discontinuation. Whether switching to another anti-TNF agent (e.g., adalimumab, golimumab) is safe remains a complex question.

Evidence is conflicting. Some reports suggest that AIHA represents a TNF-α effect, with a high risk of recurrence upon switching to another anti-TNF (13). However, other cases indicate that switching may be feasible, particularly in patients who developed AIHA later in treatment rather than early in therapy (14).

In our case, given the severity of AIHA, we opted for vedolizumab, an integrin inhibitor. This decision aligns with existing literature recommending biologics with a different mechanism of action in cases of severe autoimmune complications secondary to anti-TNF therapy (4). Given the risk of recurrence, we suggest caution when considering an intra-class anti-TNF switch and recommend close hematologic monitoring if pursued.

## Conclusion

While infliximab is an effective treatment for various autoimmune conditions, clinicians should remain vigilant for potential adverse effects, including the development of AIHA. Early recognition and appropriate management are crucial to mitigate complications associated with this serious adverse event. In our clinical scenario, the successful use of vedolizumab following infliximab-induced hemolytic anemia was an alternative and successful therapy to control IBD.

While our case suggests a possible link between infliximab and AIHA, this association remains uncertain, particularly given that AIHA can occur independently of biologic therapy in IBD patients. Although discontinuation of infliximab and initiation of corticosteroids led to clinical improvement in this patient, further research is required to determine whether infliximab plays a direct role in AIHA pathogenesis or whether this is a coincidental occurrence in the context of IBD.

In our clinical scenario, the transition to vedolizumab provided an effective alternative for disease control, supporting the idea that biologic switching can be a viable approach when managing suspected adverse immune-mediated effects of infliximab. Given that infusion reactions and infections are the most frequently observed adverse events associated with infliximab therapy, AIHA should be considered an uncommon but serious complication, necessitating careful monitoring and individualized risk assessment.

## Data Availability

The raw data supporting the conclusions of this article will be made available by the authors, without undue reservation.
